# A Bacterial Expression Vector Archive (BEVA) for Flexible Modular Assembly of Golden Gate-Compatible Vectors

**DOI:** 10.3389/fmicb.2018.03345

**Published:** 2019-01-14

**Authors:** Barney A. Geddes, Marcela A. Mendoza-Suárez, Philip S. Poole

**Affiliations:** Department of Plant Sciences, University of Oxford, Oxford, United Kingdom

**Keywords:** Golden Gate, modular assembly, cloning vector, shuttle vector, broad-host range plasmid, open source

## Abstract

We present a Bacterial Expression Vector Archive (BEVA) for the modular assembly of bacterial vectors compatible with both traditional and Golden Gate cloning, utilizing the Type IIS restriction enzyme Esp3I, and have demonstrated its use for Golden Gate cloning in *Escherichia coli*. Ideal for synthetic biology and other applications, this modular system allows a rapid, low-cost assembly of new vectors tailored to specific tasks. Using the principles outlined here, new modules (e.g., origin of replication for plasmids in other bacteria) can easily be designed for specific applications. It is hoped that this vector construction system will be expanded by the scientific community over time by creation of novel modules through an open source approach. To demonstrate the potential of the system, three example vectors were constructed and tested. The Golden Gate level 1 vector pOGG024 (pBBR1-based broad-host range and medium copy number) was used for gene expression in laboratory-cultured *Rhizobium leguminosarum*. The Golden Gate level 1 vector pOGG026 (RK2-based broad-host range, lower copy number and stable in the absence of antibiotic selection) was used to demonstrate bacterial gene expression in nitrogen-fixing nodules on pea plant roots formed by R. *leguminosarum*. Finally, the level 2 cloning vector pOGG216 (RK2-based broad-host range, medium copy number) was used to construct a dual reporter plasmid expressing green and red fluorescent proteins.

## Introduction

Over the last decade, synthetic biology has emerged as a powerful tool for biotechnology and basic science research (Church et al., 2014). Coupled with rapidly improving DNA synthesis technologies, one of the key advances that has potentiated the rapid expansion of synthetic biology is new DNA assembly technologies (Kosuri and Church, 2014). The ability for synthetic biologists to rapidly assemble and test new designs at low cost is invaluable to progress in a wide-range of life sciences research. Applications for this range from investigating new antibody production with higher success rates, to increased food production minimizing the carbon footprint with the effective transfer of nitrogen fixation between bacterial species (Smanski et al., 2014).

Golden Gate cloning is a DNA assembly technology that utilizes type IIS restriction enzymes to assemble multiple fragments of DNA in a linear order (Weber et al., 2011). Since type IIS restriction endonucleases cleave outside of their recognition site, the nucleotides in the cut-site of the enzyme are not discriminated and can be tailored to suit. This permits assembly of multiple fragments of DNA in a directional, linear order by using unique cut/ligation sites between each fragment, all of which can be cut by the same type IIS restriction enzyme. The two most commonly used Golden Gate enzymes are BsaI and BpiI, each has a 6 base-pair (bp) recognition site and a 4 bp sticky-end cut site (Weber et al., 2011). The ability of these enzymes to cleave outside of their recognition site and careful design of compatible overhanging sequences allows repeated rounds of cutting followed by ligation to proceed toward a stable product which remains undigested because it lacks enzyme recognition sites (due to the inward orientation of the type IIS recognition sites at the far 5′ and 3′ ends of modules) (Engler et al., 2009). This approach has given a massive improvement in the efficiency of assembly compared to traditional rounds of restriction/ligation cloning and has been used to assemble at least nine fragments in linear order, with 90% of transformed bacterial colonies containing correct products (Engler et al., 2009).

Golden Gate cloning platforms have been developed for plants, fungi and the bacterium *Escherichia coli* (Engler et al., 2014; Terfrüchte et al., 2014; Agmon et al., 2015; Moore et al., 2016). However, in a wide-range of bacteria, vectors for cloning using Golden Gate tools are not available. The ability to use the most up-to-date molecular tools will be crucially important for the future agricultural biotechnology required to feed a growing population. Here, we describe the development of a system for modular assembly of Golden Gate-compatible cloning broadhost range vectors from a library of vector parts. Vectors are assembled in *E. coli* and can be transferred easily to other bacterial species by conjugation. A key advantage of the tool kit described herein is the development of Golden Gate compatible plasmids with broad host range for use in Gram-negative bacteria phylogenetically distinct from *E. coli*. We demonstrate the efficacy of this system by constructing several vectors, analogous to useful traditional cloning broad-host range vectors but that are now Golden Gate-compatible: level 1 cloning broad-host range vectors pOGG024, a medium copy plasmid for bacterial gene, and pOGG026, a low copy number vector for gene expression in environmental samples where no antibiotic selection is applied. We also describe the construction through this modular vector system of a level 2 broad-host range vector pOGG216.

## Results and Discussion

### Modular Vector Assembly

A new standardized system for vector assembly was designed based on Golden Gate cloning and the MoClo system described by Weber et al. (2011). To describe cloning into vectors constructed by this system we maintain the defined hierarchical assembly language in MoClo. “Level 0” refers to base parts that make up an open reading frame (ORF; ex: promoter, coding sequence, and terminator), “level 1” refers to a single ORF made up of level 0 parts, and “level 2” refers to several ORFs organized together (Weber et al., 2011). Level 1 cloning describes assembly of level 0 parts into an ORF using the enzyme BsaI. Level 2 cloning describes assembly of level 1 ORFs into a multi-gene construct using BpiI. A level 1 cloning vector is used for assembly of a single ORF by level 1 cloning. A level 2 cloning vector is used for the assembly of multiple ORFs together by level 2 cloning (Weber et al., 2011).

**Figure 1:**
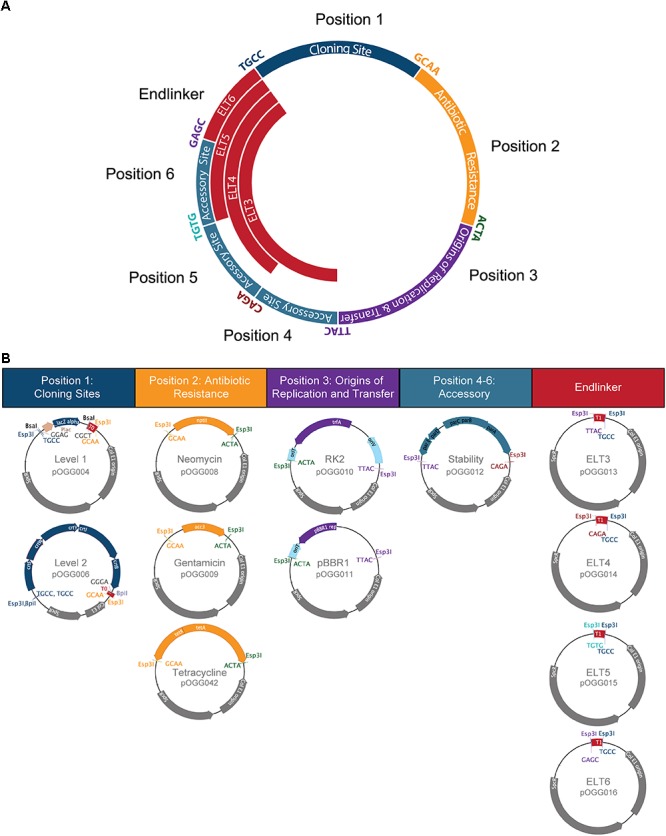
Bacterial Expression Vector Archive (BEVA) system for the modular assembly of bacterial vectors. **(A)** Vector modules are shown in the order they are assembled in a vector construction reaction. Their position in the final construct is labeled. Sequences (5′→ 3′) used to anneal the fragments in order are shown (color-coded) at junctions between modules and remain in the final construct as a scar. **(B)** Modules at each position. Position 1: cloning sites – Golden Gate level 1 and Golden Gate level 2; position 2: antibiotic resistance – neomycin-, gentamicin- and tetracycline-resistance cassettes; position 3: origins of replication and transfer – RK2 with *oriT* and pBBR1 with *oriT*; positions 4–6: accessory modules – *par* locus for stable plasmid maintenance in the absence of antibiotic selection. Endlinker modules were designed to circularize the plasmid.

We have designed the bacterial vector assembly system with a series of discrete key modules. In order to do this, we took advantage of the modularization and miniaturization of bacterial vector modules in the Standard European Vector Architecture (SEVA) (Silva-Rocha et al., 2013; Martínez-Garćía et al., 2015). In SEVA, these modules are arranged in a predefined order and assembled using standard restriction enzymes and standard restriction/ligation reactions. We have conserved this organization in the modular vector assembly system: position 1 is the cloning site(s), position 2 is the antibiotic resistance cassette, position 3 is origins of replication and transfer. Three more positions (4 to 6) have been reserved for additional accessory modules for specific applications. Endlinkers containing terminators (ELT) are used to circularize the plasmid by connecting the final position used to position 1 (Figure 1A). We incorporated Golden Gate level 1 and level 2 cloning sites into position 1 to make them compatible with the MoClo Golden Gate cloning system (Figure 1B). Each vector construction module is flanked with inward-facing recognition sites for the type IIS restriction enzyme Esp3I and the cut sites, giving overhangs with sequences specific for each module (fusion sites shown in Table 1), allow directional linear assembly in a one-pot reaction such as used in Golden Gate cloning (Weber et al., 2011).

**Table 1:** Design of modules for vector construction.

Module	Description of module	Module size (kb)	Cloned module	5′-side (5′→3′)	3′-side (5′→3′)
**Position 1: cloning sites**				TGCC	GCAA
pLVC-P1-Lv1	Golden Gate Level 1 cloning site with cloned *lacZ*	2601	pOGG004		
	Golden Gate Level 1 cloning site	2420	pOGG005		
pLVC-P1-Lv2	Golden Gate Level 2 cloning site	5729	pOGG006		
**Position 2: antibiotic resistance**				GCAA	ACTA
pLVC-P2-neo	Neomycin/kanamycin-resistance	936	pOGG008		
pLVC-P2-gent	Gentamicin-resistance	813	pOGG009		
pLVC-P2-tet	Tetracycline-resistance	1964	pOGG042		
**Position 3: origins of replication and transfer**				ACTA	TTAC
pLVC-P3-RK2	RK2 (IncP) origin of replication for low copy number plasmid, *oriT* from *E. coli* for plasmid transfer	2485	pOGG010		
pLVC-P3-pBBR1	pBBR1 origin of replication for medium copy number plasmid, *oriT* from *E. coli* for plasmid transfer	1784	pOGG011		
**Position 4: accessory**				TTAC	CAGA
pLVC-P4-par	Partition genes (*parABCDE*) for plasmid stability in absence of antibiotic selection	2408	pOGG012		
**Position 5: accessory**				CAGA	TGTG
**Position 6: accessory**				TGTG	GAGC
**Endlinker**					
pLVC-ELT-3	Endlinker to circularize plasmid after position 3	113	pOGG013	TTAC	TGCC
pLVC-ELT-4	Endlinker to circularize plasmid after position 4	113	pOGG014	CAGA	TGCC
pLVC-ELT-5	Endlinker to circularize plasmid after position 5	113	pOGG015	TGTG	TGCC
pLVC-ELT-6	Endlinker to circularize plasmid after position 6	113	pOGG016	GAGC	TGCC

### Vector Module Design

Modules at each position (1 to 6 and Endlinkers, Table 1) have inward-facing Esp3I sites which on digestion give 4 bp-overhangs. These overhangs are able to anneal with those from neighboring modules in a linear fashion and, on vector assembly, give 4 bp motifs which remain at the join between modules (all motifs for each junction are listed in Table 1 and Figure 1). An advantage of modular assembly is the ability to utilize minimal parts which exclude any non-functional DNA. This allows construction of vectors with the same key features as those currently widely adopted, but the resulting plasmid is significantly smaller in size. The reduced size enhances the ease of DNA manipulation and allows accommodation of larger cloned inserts. To this end, several modules (positions 2 and 3) used the minimal units of antibiotic-resistance modules and origins of replication/transfer previously defined by SEVA (Silva-Rocha et al., 2013; Martínez-Garćía et al., 2015; Table 1).

Most parts were constructed by DNA synthesis by Invitrogen and supplied in the GeneArt cloning vector pMS (Invitrogen, Thermo Fisher Scientific Inc.). As an alternative, some vector modules were constructed by PCR amplification from commonly used vectors (see Table 2). In several cases, this was a cheaper and faster option than the synthesis of the DNA fragments. Modules synthesized by Invitrogen were domesticated prior to DNA synthesis by removal of BsaI, BpiI, DraIII, and Esp3I (Golden Gate cloning restriction enzyme sites) by altering a single nucleotide in the recognition site. In an ORF, a neutral change was made in the wobble base of a codon, such that the same amino acid was encoded. Outside of an ORF, a transition mutation was introduced.

**Table 2:** Level 0 modules for cloning in a Level 1 vector.

Module	Description of module	Module size (bp)	Cloned module	5′-side (5′ → 3′)	3′-side (5′ → 3′)
**PU modules: promoters with ribosome-binding sites**				GGAG	AATG
pL0M-PU-pNeo	Promoter upstream of *nptII* for constitutive gene expression	386	pOGG001		
pL0M-PU-pT7lacO	IPTG-inducible T7RNAP promoter	95	pOGG030		
pL0M-PU-pLac	IPTG-inducible promoter	258	pOGG031		
pL0M-PU-pTau	Taurine-inducible promoter for Alphaproteobacteria	1811	pOGG041		
pL0M-PU-pNifH	Promoter upstream of *nifH* for nodule-specific gene expression in *R. leguminosarum*	721	pOGG082		
**SC modules: ORFs**				AATG	GCTT
pL0M-SC-mCherry	Red fluorescence protein mCherry, reporter of gene expression	716	EC15071		
pL0M-SC-sfGFP	Superfolder GFP, stable and bright reporter of gene expression	722	pOGG037		
pL0M-SC-celB	Thermostable beta-galactosidase, reporter of gene expression	1424	pOGG050		
pL0M-SC-gusA	Beta-glucosidase gene, reporter of gene expression	1817	pOGG083		
**T modules: terminators**				GCTT	CGCT
pL0M-T-pharma	Pharmacia terminator (Invitrogen)	188	pOGG003		
pL0M-T-T7	Terminator for T7RNAP	55	pOGG039		

*All modules were supplied cloned in Sp^*r*^ plasmid pMS except for pOGG082 and pOGG083. Following BsaI digestion of cloned modules and a Level 1 vector, the resulting overhangs are used to anneal the fragments in a linear fashion and the sequences shown remain between modules upon ligation*.

The position 1 modules we designed are based on level 1 and level 2 cloning sites pL1F-1 and pL2V-1 from the MoClo system of Weber et al. (2011) (Table 1). The level 1 cloning site in pOGG004 contains a *lacZα* fragment that is replaced following Golden Gate cloning in the MoClo system, resulting in blue to white colony color selection when plated on appropriate media. Since *lacZα* still contains a polylinker, vectors constructed with the level 1 Golden Gate cloning site are also flexible for use in traditional cloning utilizing unique restriction sites in the polylinker and blue-white selection. The level 2 Golden Gate cloning site encodes a canthaxanthin biosynthesis operon that is replaced upon successful level 2 cloning resulting in a transition from an orange colony color to white or blue. Both position 1 modules (level 1 and level 2 cloning sites) include a *rho*-independent T_0_ lambda phage transcription terminator (Lutz and Bujard, 1997) included at their 3′-end.

We note that the level 1 Golden Gate cloning site part (pOGG004) is maintained in a high copy number vector backbone suitable for recombinant protein production in *E. coli*. Thus it can be utilized as a level 1 cloning vector for this purpose. To expand this utility we also synthesized a version with an ampiciliin-resistant backbone (pOGG005).

For position 2 (antibiotic resistance), gentamicin- and neomycin-resistance modules were based on the minimal resistance cassettes from SEVA (Silva-Rocha et al., 2013). The 5′-end SwaI and 3′-end PshAI sites that flanked the SEVA modules were replaced with inward-facing Esp3I sites. We found that the SEVA constitutive *tetA* gene conferred insufficient levels of tetracycline resistance to our organisms of interest (data not shown), therefore we designed a more robust tetracycline-resistance module based on the *tetAR* resistance module from pJP2 (Prell et al., 2002). To preserve a minimal size, only DNA between the stop codons of the divergently transcribed *tetA* and *tetR* was used and their orientation conserved (Figure 1B). In position 3 (origins of replication and transfer), modules containing pBBR1 and RK2 origins of replication were designed based on SEVA modules (Silva-Rocha et al., 2013), but with inward-facing Esp3I sites replacing PshAI and AscI sites. An origin of transfer (*oriT*), to permit conjugation from *E. coli* into target strains, was included at the 5′-end. At position 4, an accessory module for stable plasmid maintenance in the absence of antibiotic selection was designed. Based on the *par* locus from pJP2, which has previously been shown to render stable plasmid maintenance in the absence of antibiotic selection (Prell et al., 2002), *parABCDE*, as well as 107 bp downstream of *parE* (at 5′-end) and 139 bp downstream of *parA* (at 3′-end) (Figure 1B) was oriented such that the *parE* was transcribed toward position 3, while *parA* was at the 3′-end, transcribed toward position 1 (Figure 1B).

Endlinker (ELT) modules were designed to complete assembly by circularizing the plasmid, either by linking position 3 (origin of replication), or accessory modules at positions 4, 5, or 6 to position 1 (cloning sites). Endlinker modules contain the *E. coli* T_1_ *rrnBT1* terminator to help buffer any transcription effects from the cloning site (Lutz and Bujard, 1997).

### Design and Construction of Level 0 Open Reading Frame Parts

Golden Gate cloning is often used in a hierarchical assembly where level 0 parts (genetic features controlling a gene’s transcription, translation and the termination of transcription) are assembled in a single one-pot reaction wherein parts are added as plasmids together with restriction enzyme and ligase in a single tube (referred to as level 1 assembly) allowing directional linear assembly (Weber et al., 2011). In order to test the vectors that we constructed, we adapted several parts that are routinely used in our lab, including promoters with ribosome binding sites (PU modules – **p**romoter and **u**ntranslated region), ORFs (SC modules – **s**ignal and **c**oding sequence) and a terminator (T module). These parts were synthesized as described for vector construction modules, except that they were assembled with inward-facing BsaI sites that cut appropriate sequences for level 1 assembly (Table 2).

We adapted several promoters routinely used for gene expression in rhizobia or *E. coli* for use as PU modules (Table 2). These are the constitutive neomycin cassette promoter (pNptII) (Frederix et al., 2014) T7RNAP promoter repressible by LacI (pT7lacO) (Berrow et al., 2009), the Lac promoter from *E. coli* (*pLac*) (Keen et al., 1988), the taurine-inducible promoter of *S. meliloti* (*pTau*) (Tett et al., 2012) and the symbiosis-induced *nifH* promoter from *Rhizobium leguminosarum* biovar *viciae* 3841 (Rlv3841P*nifH*) (Johnston and Beringer, 1975). For all promoters, the nucleotide immediately upstream of the ATG was domesticated to an A to construct the 5′-AATG-3′ cut-site such that the ATG encodes the start codon of the ORF to be expressed.

For SC modules (Table 2), we utilized several different reporter genes to demonstrate vector function. These include genes encoding superfolder green fluorescent protein (sfGFP) (Pédelacq et al., 2006), *E. coli* β-glucuronidase (*gusA*) and *Pyrococcus furiosus* thermostable β-glucosidase/ β-galactosidase (*celB*) (Sessitsch et al., 1996; Sánchez-Cañizares and Palacios, 2013). Domestication and gene synthesis were used to produce SC modules of sfGFP and *celB*, and *gusA*. All SC modules were constructed flanked by inward facing BsaI restriction site cutting 5′-AATG-3′ at the 5′ end and GCTT at the 3′ end. In all cases the final three nucleotides of the AATG scar encode the start codon for the ORF and the GCTT scar immediately follows the stop codon.

For use as a transcriptional terminator, the *rrnBT1* transcriptional terminator with flanking regions from pLMB509 (Tett et al., 2012) (9 bp upstream and 84 bp downstream) were synthesized and flanked by BsaI sites cutting GCTT and CGCT on the 5′ and 3′ of the ORF. All the novel BEVA parts, vectors and plasmids are available through Addgene (Addgene ID^1^ is shown in Table 3).

**Table 3:** Strains, plasmids, and primers used in this study.

Strain, plasmid or primer	Description	Source or reference	Addgene ID
**Strain**			
*E. coli* strains			
α-Select Gold	Competent cells. Genotype: F – deoR endA1 recA1 relA1 gyrA96 hsdR17(rk-, mk + ) supE44 thi-1 phoA Δ(lacZYA argF)U169 Φ80lacZΔM15λ -	Bioline	
DH5α	Competent cells. Genotype: F- Φ80lacZΔM15 Δ(lacZYA-argF) U169 recA1 endA1 hsdR17(rk-, mk+) phoA supE44 thi-1 gyrA96 relA1 λ-	Invitrogen	
*R. leguminosarum* bv. *viciae* 3841	*Rhizobium leguminosarum* bv. *viciae*, derivative of strain 300, Str^r^	Johnston and Beringer, 1975	
*Rhizobium tropici* CIAT899	*Rhizobium tropici*, Rf^r^	Graham et al., 1982	
**Plasmid**			
pJP2	pTR102 GUS with artificial MCS, Amp^r^ Tet^r^	Prell et al., 2002	
EC15071	pL0M-SC-mCherry. Level 0 SC module cloned in pMS, Sp^r^	ENSA, supplied by Invitrogen	
pMS	Vector with ColE1 origin of replication in which modules are supplied from Invitrogen, Sp^r^	Invitrogen	
pLMB509	Highly inducible His tag expression vector, Gm^r^	Tett et al., 2012	40084
pOGG001	pL0M-PU-pNeo, promoter 379 bp upstream from of the start codon of luxC from pIJ11268 of nptII for constitutive gene expression. Level 0 PU module cloned in pMS, Sp^r^	This study	113978
pOGG003	pL0M-T-pharma. Level 0 T module cloned in pMS, Sp^r^	Invitrogen	
pOGG004	pLVC-P1-Lv1 (Golden Gate Level 1 cloning site with cloned lacZ) position 1 module for vector construction cloned in pMS, Sp^r^	This study	113979
pOGG005	pL1V-Lv1-amp-ColE1, Level 1 cloning vector, high copy number used for protein expression in *E. coli*, Amp^r^	This study	113980
pOGG006	pLVC-P1-Lv2, Golden Gate Level 2 cloning site, position 1 module for vector construction cloned in pMS, Sp^r^	This study	113981
pOGG008	pLVC-P2-neo, neomycin-resistance gene, position 2 module for vector construction cloned in pMS, Spr Nm^r^/Kan^r^	This study	113982
pOGG009	pLVC-P2-gent, gentamicin-resistance gene, position 2 module for vector construction cloned in pMS, Sp^r^ Gm^r^	This study	113983
pOGG010	pLVC-P3-RK2, RK2 origin of replication and oriT from *E. coli*, position 3 module for vector construction cloned in pMS, Sp^r^	This study	113984
pOGG011	pLVC-P3-pBBR1, pBBR1 origin of replication and oriT from *E. coli*, position 3 module for vector construction cloned in pMS, Sp^r^	This study	113985
pOGG012	pLVC-P4-par, partition genes (parABCDE from pMS) for plasmid stability, position 4 module for vector construction cloned in pMS, Sp^r^	This study	113986
pOGG013	pLVC-ELT-3, connecting position 3 to position 1 to circulaise vector, endlinker module for vector construction cloned in pMS, Sp^r^	This study	113987
pOGG014	pLVC-ELT-4, connecting position 4 to position 1 to circulaise vector, endlinker module for vector construction cloned in pMS, Sp^r^	This study	113988
pOGG015	pLVC-ELT-5, connecting position 5 to position 1 to circulaise vector, endlinker module for vector construction cloned in pMS, Sp^r^	This study	113989
pOGG016	pLVC-ELT-6, connecting position 6 to position 1 to circulaise vector, endlinker module for vector construction cloned in pMS, Sp^r^	This study	113990
pOGG021	Destination vector for pL1V-F1, Amp^r^	Weber et al., 2011	
pOGG024	pL1V-Lv1-gent-pBBR1-ELT3, 3.4 kb, medium copy, broad-host range Level 1 cloning vector, Gm^r^	This study	113991
pOGG026	pL1V-Lv1-neo-RK2-par-ELT4, 6.6 kb, low copy, environmentally stable, broad-host range Level 1 cloning vector, Nm^r^/Kan^r^	This study	113992
pOGG030	pL0M-PU-pT7lacO, IPTG-inducible T7RNAP promoter 88 bp promoter and RBS driving recombinant protein expression in the pET and pOPIN vectors. Level 0 PU module cloned in pMS, Sp^r^	This study	113993
pOGG031	pL0M-PU-pLac, IPTG-inducible promoter 250 bp immediately upstream of the lacZ alpha fragment start codon in pRK415. Level 0 PU module cloned in pMS, Sp^r^	This study	113994
pOGG037	pL0M-SC-sfGFP, pMS Level 0 SC module cloned in pMS, Sp^r^	This study	113995
pOGG039	pL0M-T-T7. Level 0 T module cloned in pMS, Sp^r^	This study	113996
pOGG041	pL0M-PU-pTau, Taurine-inducible promoter for Alphaproteobacteria, Level 0 PU module cloned in pMS, Sp^r^	This study	113997
pOGG042	pLVC-P2-tet, tetracycline-resistance gene (tetAR) from pJP2. Made by PCR using oxp0734 and oxp0735 primers in position 2 module for vector construction cloned in pMS, Sp^r^, Tet^r^	This study	113998
pOGG050	pL0M-SC-celB. Level 0 SC module cloned in pMS, Sp^r^	This study	113999
pOGG054	Destination vector for pL1V-F2, Amp^r^	Weber et al., 2011	
pOGG056	Destination vector for Level 2 Endlinker ELB-2, Amp^r^	Weber et al., 2011	
pOGG068	Destination vector for pL0V-PU, Sp^r^	Weber et al., 2011	
pOGG072	Destination vector for pL0V-SC, Sp^r^	Weber et al., 2011	
pOGG082	pL0M-PU-*pNifH*, promoter 714 bp upstream of the ATG of *nifH* for nodule-specific gene expression in *R. leguminosarum.* Made by PCR using oxp0474 and oxp0475 primers and cloned in destination vector pOGG068, Sp^r^	This study	114000
pOGG083	pL0M-SC-gusA, *gusA* gene from pJP2. Made by PCR using oxp0376 and oxp0377 primers and cloned in destination vector pOGG072. Sp^r^	This study	114001
pOGG202	pL1M-F1-plac (pOGG031), sfGFP (pOGG037) and T-pharma (pOGG003), Amp^r^	This study	115503
pOGG203	pL1M-F2-pNeo (pOGG001), mCherry (EC15071) and T-pharma (pOGG003), Amp^r^	This study	115504
pOGG216	pL2V-L2-tet-pBBR1-ELT3, 9.5 kb, medium copy, broad-host range. Level 2 cloning vector, Tet^r^	This study	114002
pOPS0253	Reporter plasmid constructed with Rlv3841P*nifH* (pOGG082), *gusA* (pOGG083) and T-pharma (pOGG003) assembled in pOGG026, Nm^r^/Kan^r^	This study	115505
pOPS0254	Reporter plasmid constructed with Rlv3841P*nifH* (pOGG082), *celB* (pOGG050) and T-pharma (pOGG003) assembled in pOGG026, Nm^r^/Kan^r^	This study	115506
pOPS0314	Reporter Plasmid constructed with constitutive promoter pNeo (pOGG001), *celB* (pOGG050) and T-pharma (pOGG003) assembled in pOGG026, Nm^r^/Kan^r^	This study	115507
pOPS0359	Reporter plasmid constructed with pTau (pOGG041), sfGFP (pOGG037) and T-pharma (pOGG003) assembled in pOGG024, Gm^r^	This study	115508
pOPS0377	Reporter Plasmid constructed with constitutive promoter pNeo (pOGG001), sfGFP (pOGG037) and T-pharma (pOGG003) assembled in pOGG026, Nm^r^/Kan^r^	This study	115509
pOPS0379	Reporter plasmid constructed with Rlv3841P*nifH* (pOGG082) sfGFP (pOGG037) and T-pharma (pOGG003) assembled in pOGG026, Nm^r^/Kan^r^	This study	115510
pOSP0750	Reporter Plasmid constructed with constitutive promoter pNeo (pOGG001) *gusA* (pOGG083) and T-pharma (pOGG003) assembled in pOGG026, Nm^r^/Kan^r^	This study	115511
pOPS0754	Dual reporter plasmid. Forward position 1 pOGG202, forward position 2 pOGG203 and level 2 Endlinker ELB-2 (pOGG056) assembled in pOGG216, Tet^r^	This study	115512
**Primer**			
oxp0376	Forward primer for amplification of *gusA* gene for SC module. Sequence: CACTCTGTGGTCTCAAATGGTCCGTCCTGTAG	This study	
oxp0377	Reverse primer for amplification of *gusA* gene for SC module. Sequence: CACTTCGTGGTCTCAAAGCTCATTGTTTGCCTCCC	This study	
oxp0734	Forward primer for amplification of tetracycline-resistance gene (tetAR) from pJP2. Used in position 2 module for vector construction. Sequence: TTTTGAAGACAAGAATACAGTCATAAGTGCGGC	This study	
oxp0735	Reverse primer for amplification of tetracycline-resistance gene (tetAR) from pJP2. Used in position 2 module for vector construction. Sequence: TTTTTGAAGACAATGCCGGTCTCCATAACCGGA	This study	
oxp0474	Forward primer for amplification of P*nifH* region of Rlv3841 plasmid pRL10 for PU module. Sequence: CACTCTGTGGTCTCAGGAGTCGATGCTGACCGCCT	This study	
oxp0475	Reverse primer for amplification of P*nifH* region of Rlv3841 plasmid pRL10 for PU module. Sequence: CACTTCGTGGTCTCACATTTTTGGCGTTCCTTCATGTGT	This study	

*Amp^*r*^, ampicillin resistance; Gm^*r*^, gentamicin resistance; Kan^*r*^, kanamycin resistance; Nm^*r*^, neomycin resistance; Rf^*r*^, rifampicin resistance, Sp^*r*^, spectinomycin resistance; Str^*r*^, streptomycin resistance; Tet^*r*^, tetracycline resistance*.

### Vectors for Golden Gate Level 1 Cloning in Bacteria

Vector assembly reactions were performed by selecting appropriate modules in each position (Figure 1, summary of the sequences used to combine the modules which remain at their junctions and fusion sites are given in Table 1).

**Figure 2:**
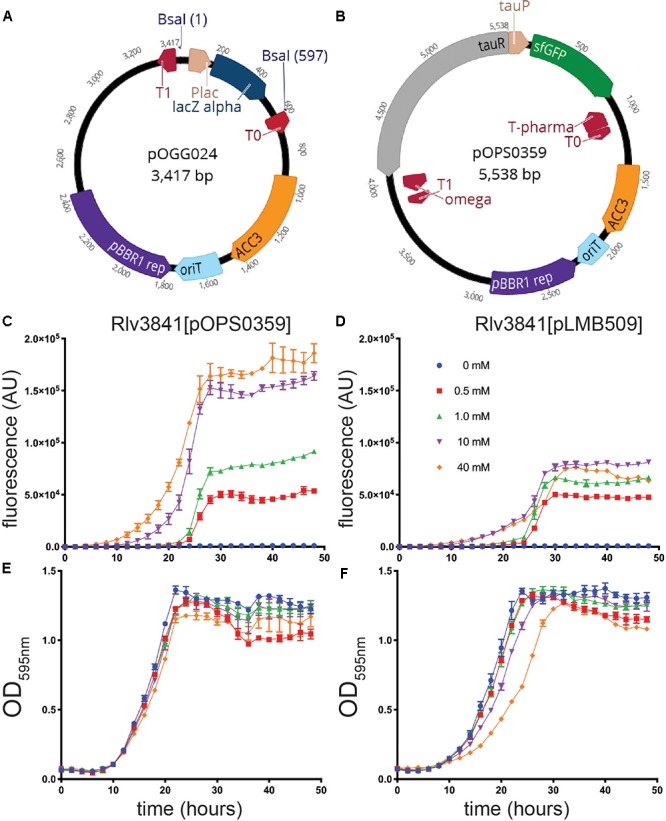
**(A)** Schematic map of pOGG024 level 1 broad-host range, medium copy number vector constructed with BEVA for free-living gene expression in laboratory. **(B)** Schematic map of plasmid pOPS0359 containing a cloned gene sfGFP under the control of a taurine-inducible promoter from *Sinorhizobium meliloti* in pOGG024. **(C–F)** Evaluation of pOPS0359 performance as robust free-living gene expression compared to the functionally analogous pLMB509 plasmid. **(C,D)** Fluorescence detection from GFP expression and **(E,F)** fitness performance by measurement of OD_595_ when grown without (0 mM) or with (0.5, 1.0, 10, or 40 mM) taurine. For all graphs, error bars are standard error of the mean (SEM).

To demonstrate the flexibility of the system, two vectors for level 1 cloning, analogous to those used for traditional cloning and DNA manipulation in Gram-negative bacteria, were constructed. Plasmid pOGG024 (Figure 2A and Table 3), a 3.4 kb level 1-compatible vector for bacterial gene expression, was made by adding the plasmids (listed in Table 3) to a one-pot reaction (digestion with Esp3I and ligation) to combine the following modules: position 1; level 1 cloning site from pOGG004, position 2; gentamicin resistance cassette from pOGG009, position 3; pBBR1-based origin of replication from pOGG011 and the appropriate Endlinker (ELT3) from pOGG013 (Table 1).

**Figure 3:**
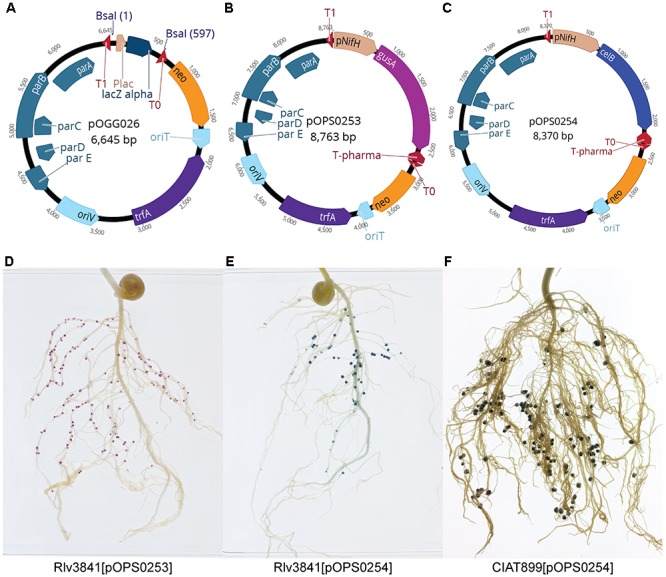
**(A)** Schematic map of pOGG026 level 1 broad-host range, lower copy number vector constructed with BEVA for stable environmental gene expression in the absence of antibiotic selection. **(B,C)** Schematic map of plasmids containing a cloned **(B)** *gusA* (pOPS0253) and **(C)** *celB* (pOPS0254) under the control of *nifH* gene promoter (P*nifH*). **(D)** Pea nodules formed by Rlv3841[pOPS253] stained with Magenta-glucA. Marker gene *gusA* is just expressed in nodules. **(E)** Pea nodules formed by Rlv3841[pOPS254] and **(F)** bean nodules formed by CIAT899[pOPS254] stained with X-gal after thermal treatment. Marker genes *gusA* and *celB* are just expressed in nodules.

Plasmid pOGG026 (6.6 kb, Figure 3A and Table 3) was constructed by combining the following modules (Table 2) in a one-pot reaction (digestion with Esp3I and ligation): position 1; level 1 cloning site from pOGG004, position 2; neomycin resistance gene from pOGG008, position 3; RK2-based origin of replication from pOGG010, position 4; *parABCDE* genes which confer stability in the absence of antibiotics from pOGG012 and the appropriate Endlinker (ELT4) from pOGG014 (Table 1).

### Gene Expression Using Vector pOGG024

In previous work, we described the construction of pLMB509 (Tett et al., 2012), a plasmid which has a taurine-inducible promoter upstream of a gene encoding GFP (*gfpmut3.1*). To evaluate the performance of vector pOGG024 through comparison with the fully-characterized pLMB509, we assembled plasmid pOPS0359 by cloning a taurine-inducible promoter upstream of superfolder GFP (sfGFP) to create a functionally analogous plasmid, i.e., taurine-inducible expression of GFP (Figure 2B). Taurine-dependent GFP expression of plasmids pOPS0359 and pLMB509 in a Rlv3841 background is shown in Figures 2C,D. Under conditions of 0 to 40 mM taurine they showed similar growth (Figures 2E,F) and gave fluorescence profiles indicating induction of GFP throughout the exponential phase. Although similar GFP induction profiles were seen, pOPS0359 showed significantly higher levels of overall fluorescence, especially at 10 and 40 mM taurine. As the plasmids share the same origin of replication (pBBR1) there are several factors that are likely to have had an influence on this; the brighter fluorescence of sfGFP (Pédelacq et al., 2006) (pOPS0359) compared to that of GFPmut3.1 (pLMB509), and the reduced size of pOPS0359 (5.5 kb) compared to pLMB509 (6.8 kb) perhaps leading to an increased plasmid copy number. Alternatively, codon preference in the nucleotide sequence may have influenced expression levels as neither sequences was codon optimized for Rlv3841.

### Gene Expression Using pOGG026

To demonstrate the flexibility of BEVA we constructed a relatively low copy number, stable plasmid pOGG026, suitable for experiments in environments where there is no antibiotic selection present.

**Figure 4:**
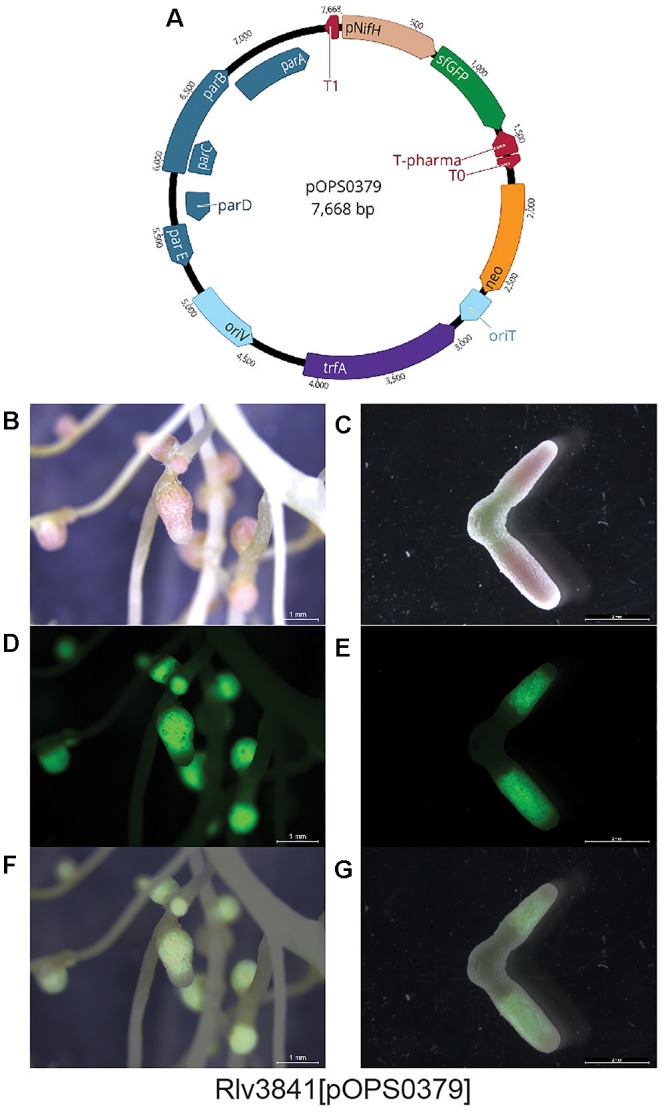
**(A)** Schematic map of plasmid pOPS0379 containing sfGFP gene under the control of *nifH* gene promoter (P*nifH*). Pea nodules formed by Rlv384[pOPS0379], **(B,C)** bright-field, **(D,E)** fluorescence (GFP filter), and **(F,G)** merged images. GFP expression is observed in the nitrogen-fixing zone.

To demonstrate the stability of pOGG026, and therefore its use for experiments carried out in many different environments, it was used to construct plasmids pOPS0253 (Figure 3B), pOPS0254 (Figure 3C) and pOPS0379 (Figure 4A) by Golden Gate cloning. These plasmids were conjugated into *R. leguminosarum* Rlv3841 and used to inoculate peas. Plasmid pOPS0254 was also conjugated into R. *tropici* CIAT 899 (Graham et al., 1982) and used to inoculate bean plants. We did five biological replicates of all the inoculations.

The expression of the chromogenic marker genes in plasmids pOPS0253 and pOPS0254 can be followed by staining either pink (*gusA*) or blue (*celB*) when the appropriate substrate is supplied (Sessitsch et al., 1996). Stained pea nodules are shown in Figures 3D,E and stained bean nodules are shown in Figure 3F. Figures 4B,C show pea nodules formed by Rlv3841[pOPS0379]. Figure 4B shows mature nodules 28 dpi and Figure 4C shows senescent nodules 42 dpi with the senescent area furthest from the tip. The inducible expression of sfGFP can be observed when nodules reach mature stage (Figure 4D) and just in the nitrogen fixing zone (Figures 4D–G). GFP fluorescence was not detected in senescent areas of nodules (Figure 4G).

In nodules plasmids are often lost and there is an inability to apply antibiotic selection to maintain those which have a plasmid-borne resistance cassette (Schmidhauser and Helinski, 1985; Weinstein et al., 1992; Corich et al., 2001). In our assays, all mature nodules showed fluorescence or corresponding staining after enzymatic reaction with Magenta-glcA or X-gal, but to further verify plasmid stability, at least three nodules from each plant were removed, surface-sterilized and crushed into 100 μl sterile distilled water. Aliquots of each nodule extract were spotted onto TY agar plates. Afterward, eight independent colonies from each crushed nodule were screened for antibiotic resistance and all colonies showed neomycin resistance, corresponding to the plasmid vector pOGG026. In total at least 120 independent colonies from nodules were screened and none were detected that had lost the plasmid.

Using pOPS0253 and pOPS0254 we have developed a plasmid-based system that can be utilized for assessing competitiveness between rhizobia which is similar to the chromosomally encoded strategy described by Sánchez-Cañizares and Palacios (2013). We note that pOPS0379 could be used in a non-invasive symbiosis-induced screening system. Marked rhizobia bearing the plasmid could be inoculated onto plants in a mixed rhizobial population, identified by fluorescence, and recovered live from nodules for further analysis.

### Golden Gate Level 2 Cloning Vectors

**Figure 5:**
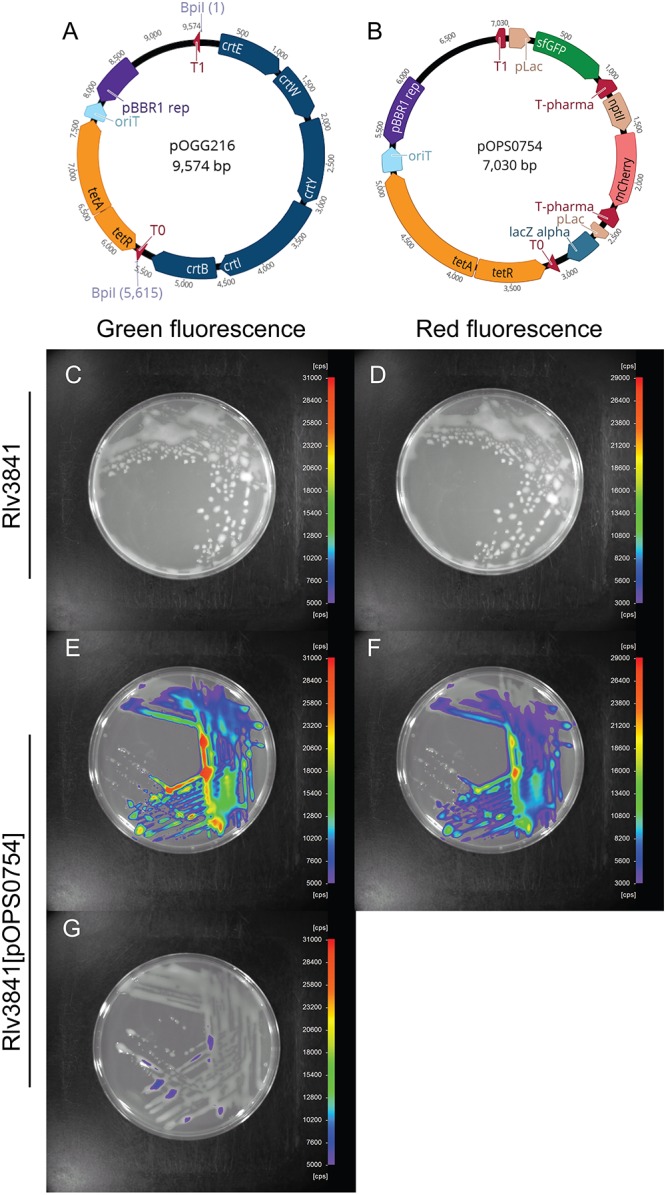
**(A)** Schematic map of pOGG216 level 2 broad-host range, medium copy number vector constructed with BEVA for multi-gene expression. **(B)** Schematic map of dual reporter plasmid pOPS0754 which encodes IPTG-inducible sfGFP cloned in Forward Position 1 and constitutively mCherry cloned in Forward Position 2. **(C,E,G)** Green fluorescence detection (scale, 5,000–31,000). **(D,F)** Red fluorescence detection (scale, 3,000–29,000 cps). **(C,D)** Rlv3841 without fluorescence. Rlv3841[pOPS0754] expressing IPTG-inducible sfGFP grown in media **(E)** containing 0.5 mM IPTG or **(G)** without IPTG. **(F)** Rlv3841[pOPS0754] with constitutively expressed mCherry.

We designed our level 2 vectors to be directly compatible with the methods and plasmids described in the MoClo system by Weber et al. (2011) for construction of level 2 plasmids containing multiple transcriptional units. In the MoClo system, multigene constructs are constructed by assembling transcriptional units by Golden Gate level 1 cloning reaction into level 1 shuttle vectors that dictate the final position in a level 2 construct. Transcriptional units are then combined with an endlinker using a Golden Gate level 2 cloning reaction with BpiI. Endlinkers can either terminate the construct or provide cloning sites for iterative rounds of cloning (Weber et al., 2011). To demonstrate the flexibility of the assembly system we have designed and constructed a level 2 destination vector, pOGG216 (Figure 5A). Vector pOGG216 was made by a one-pot reaction for vector assembly (Esp3I and ligase) with the following modules (listed in Table 2): position 1; level 2 cloning site from pOGG006, position 2; tetracycline-resistance module from pOGG042, position 3; pBBR1 origin from pOGG011 and an appropriate Endlinker (ELT3) from pOGG013. The level 2 cloning site encodes an operon for canthaxanthin biosynthesis that is replaced upon successful level 2 cloning and allows identification of successful clones based on an orange to white colony color phenotype.

### Gene Expression Using Vector pOGG216

To validate the performance of vector pOGG216, a level 2 cloning reaction was performed (one-pot reaction with BpiI and ligase) to assemble two ORFs encoding IPTG-inducible sfGFP and constitutively expressed to construct pOPS0754 (Figure 5B), Assembly in *E. coli* of the final dual reporter plasmid pOPS0754 was very efficient (>90% correct constructs, the same as described by Weber et al. (2011). The plasmid was conjugated into Rlv3841 and the observed green and red fluorescence from this dual reporter plasmid is shown in Figure 5. Significant green and red fluorescence were observed in Rlv3841[pOPS0754] compared to the Rlv3841 wild-type (Figures 5C–F and Supplementary Figure 1). GFP fluorescence of Rlv3841[pOPS0754] was dependant on growth in the presence of IPTG (Figures 5E,G).

## Conclusion

In conclusion, the BEVA system we describe has proved to be robust and flexible for creating new bacterial vectors. The modular vectors we constructed with this system behaved consistently well in the Gram-negative bacteria tested (*E. coli* and *R. leguminosarum*), and demonstrated stability in the environments tested (rhizosphere and nitrogen-fixing root nodules formed by symbiotic bacteria) when engineered to contain the stability accessory module (*par* genes). The BEVA vectors for use in Golden Gate cloning (level 1: pOGG005, pOGG024 and pOGG026, and level 2: pOGG216) have been made available through Addgene (Table 3). While these vectors are useful for cloning and gene expression in *R. leguminosarum*, the major advantage of the modular system described here is its flexibility. The ability to rapidly assemble BEVA vectors with unique features, specifically suited to a specific purpose, e.g., alternative antibiotic resistance modules to avoid conflict with other markers, or to alter the origin of replication to avoid plasmid incompatibility has enormous applications for those working in many different areas. Rapid assembly of new BEVA vectors from such a bank of parts in this way offers a quick and economical option for construction of new vectors, not only for bacteria, as the system could be expanded to include parts for plant transformation or replication and maintenance in fungi.

## Materials and Methods

### Bacterial Media and Growth Conditions

Bacterial strains and plasmids used in this study are listed in Table 3. *E. coli* strains were grown in liquid or on solid Luria–Bertani (LB) medium (Sambrook et al., 1989) at 37°C, supplemented with antibiotics at the following concentrations: tetracycline (10 μg ml^-1^), gentamicin (10 μg ml^-1^), kanamycin (20 μg ml^-1^) or spectinomycin (100 μg ml^-1^). Rhizobial strains were grown on tryptone yeast (TY) agar or broth (Beringer, 1974) or universal minimal salts (UMS) (Poole et al., 1994) at 28°C. Antibiotics were added to TY when necessary at the following concentrations: streptomycin (500 μg ml^-1^), tetracycline (5 μg ml^-1^), gentamicin (20 μg ml^-1^), rifampicin (50 μg ml^-1^), and neomycin (40 μg ml^-1^).

Plasmids were transferred into wild-type (Rlv3841 and R. *tropici* CIAT899 backgrounds by triparental mating according to Figurski and Helinski (1979).

### Golden Gate Vector Assembly, Level 1 and Level 2 Cloning

Vector assembly was performed in a thermocycler in 15 μl total volume, containing 40 fmols of each cloned module (position or ELT) (which equates to approximately 1 μl of 4 Kb plasmid at 100 ng μl^-1^), 1.5 μl Bovine Serum Albumin (1 mg ml^-1^), 1 μl Esp3I FD (Thermo Scientific), 1 μl concentrated T4 DNA Ligase (New England Biolabs), 1.5 μl T4 DNA Ligase buffer (New England Biolabs) with water to 15 μl. Tubes were prepared on ice before 25 cycles: 3 min at 37°C then 4 min at 16°C, followed by 5 min at 50°C and 5 min at 80°C. Samples were cooled on ice and 1 μl was transformed into 10 μl of *E. coli* competent cells (Bioline Gold) by heat shock for 1 min at 42°C (Engler et al., 2008) and plated onto LB with appropriate antibiotics (for pOGG024 selection was for gentamicin-resistance, for pOGG026 for kanamycin resistance and for pOGG216 for tetracycline resistance) and X-gal at 40 μg ml^-1^. Several colonies showing the correct color, i.e., blue for pOGG024 and pOGG026 (level 1) vector assembly (color is conferred by the presence of *lacZα* in the cloning site) and orange for pOGG216 (level 2) (conferred by a canthaxanthin biosynthesis cluster in the cloning site) (Weber et al., 2011), were screened by plasmid restriction digest and sequencing to verify correct constructs. Correct plasmid assembly was observed at very high frequencies and comparable to those described for Golden Gate cloning reactions (Weber et al., 2011).

Level 1 cloning was performed as described above, except BsaI FD (Thermo Scientific) replaced Esp3I. For level 2 cloning, BpiI (Thermo Scientific) was used instead of Esp3I. Transformation was performed as described above and colonies showing the correct color based on the replacement of modules in the vector cloning sites (blue to white for level 1 and orange to blue or white for level 2) were screened by plasmid restriction digest and sequencing to verify correct constructs.

The plasmid pOPS0359 was assembled by a Golden Gate level 1 cloning reaction performed with the following components (listed in Table 2) in a one-pot reaction (digestion with BsaI and ligation): vector; pOGG024, PU module; *Sinorhizobium meliloti* taurine promoter from pOGG041, SC module; sfGFP from pOGG037, T module; T-pharma terminator from pOGG003.

The plasmid pOPS0253 was constructed from the following (listed in Table 3): vector; pOGG026, PU module; Rlv3841P*nifH* from pOGG082, SC module; *gusA* from pOGG083; T module; T-pharma terminator from pOGG003. Plasmids pOPS0254 and pOPS0379 are identical to pOPS0253, except that the SC modules are *celB* from pOGG050 and sfGFP from pOGG037, rather than *gusA*. Positive controls were constructed with the constitutive neomycin cassette promoter (pNptII) from pOGG001 (PU) instead of the Rlv3841P*nifH* from pOGG082 resulting in the plasmids.

The dual reporter plasmid pOPS0754 was constructed using the following parts in a level 2 Golden Gate cloning reaction: level 2 vector pOGG216, forward position 1 from pOGG202, forward position 2 from pOGG203 and level 2 Endlinker ELB-2 from pOGG056. Plasmid pOGG202 (clone of a level 1 unit which encodes IPTG-inducible sfGFP) was constructed by combining the following: forward position 1 shuttle vector pL1V-F1 pOGG021, PU module pLac from pOGG031, SC module sfGFP from pOGG037 together with T module T-pharma from pOGG003. Plasmid pOGG203 (clone of a level 1 unit which encodes a constitutively expressed mCherry) was assembled by combining the following: forward position 2 shuttle vector pL1V-F2 pOGG054, PU module pNeo from pOGG001, SC module mCherry from EC15071 and T module T-pharma from pOGG003.

### Plant Growth

*Pisum sativum* (pea) and *Phaseolus vulgaris* (bean) seeds were surface sterilized by immersion in 95% ethanol for 0.5 min, followed by washing with sterile water. Seeds were then immersed in 2% sodium hypochlorite for 5 min. After washing the seeds five times with sterile water, they were placed onto 1% agar (DWA) plates in the dark at room temperature for 4–5 days to allow germination. Pea seedlings were placed in sterile 1-liter pots with medium vermiculite and supplied with 400 ml nitrogen-free rooting solution (Poole et al., 1994). In the case of beans, seedlings were placed in sterile 2-liter pots with fine vermiculite and supplied with 800 ml nitrogen-free rooting solution, (Broughton and Dilworth, 1971). Plants were inoculated at approximately 10^7^ cfu of rhizobia per pot and plants without inoculation were grown as a control. Plants were grown (random positioning of pots) in a controlled growth chamber at 21°C, 16-h/8-h day/night cycle.

### Enzyme Activity in Nodules

Pea plants were harvested 21 dpi and bean plants 42 dpi. Roots were stained with Magenta-glcA (5-bromo-6-chloro-3-indolyl-β-D-glucuronide acid, 200 μg ml^-1^) for plants nodulated with strain Rlv3841[pOPS0253] containing the ß-glucuronidase gene (*gusA*). For plants nodulated with rhizobial strains Rlv3841[pOPS0254] and CIAT 899[pOPS0254] containing ß-glucosidase gene (*celB)*, roots were stained with X-gal (5-bromo-4-chloro-3-indolyl- β-D-galactopyranoside, 250 μg ml^-1^) after thermal treatment at 70°C for 30 min to destroy endogenous β-galactosidases (Sessitsch et al., 1996).

### Measurement of Bacterial Growth and GFP Assays

Measurements of bacterial growth OD_595_ and GFP fluorescence (excitation 485 nm and emission 520 nm) were performed with a FLUOstar OMEGA (Lite) photometer. Rhizobia were grown on TY agar slopes with appropriate antibiotics for 3 days, washed with UMS and resuspended in UMS with 30 mM pyruvate and 10 mM NH_4_Cl (as carbon and nitrogen sources) at an OD_595_ of 0.1 at the start of the assay. Assays were performed in 24-well clear bottom micro-titre plates with 400 μl culture. Cells were incubated at 28°C with orbital shaking at 500 RPM for 48 h with measurement of optical density and fluorescence every 30 min.

### Microscopy

Photographs were taken using a dissecting microscope (Leica M165 FC) with a Leica DFC310 FX digital camera accompanying software (LAS v4.5). Visible light, cherry and GFP filters were used.

### Image Acquisition for Green and Red Fluorescence Expression

NightOWL camera (Berthold Technologies) was used for imaging fluorescence of bacteria grown on agar plates or plant roots. Each CCD image consisted of an array 1,024 by 1,024 pixels, and after acquisition, images were postprocessed for cosmic suppression and background correction. Fluorescence CCD images were acquired with a Ring-light epi illumination accessory exposed for 1 s and special filters have to be used. Filter used for GFP quantification at excitation wavelength 475/20 and emission wavelength 520/10 nm. Filter used for mCherry quantification at excitation wavelength 550/10 and emission wavelength 620/10 nm. Images were analyzed with the imaging software IndiGO (Berthold Technologies).

The author responsible for distribution of materials integral to the findings presented in this article is: Philip S. Poole (philip.poole@plants.ox.ac.uk).

## Author Contributions

BG conceived the idea. BG and MM-S carried out the experiments and wrote the manuscript. PP supervised the project.

## Conflict of Interest Statement

The authors declare that the research was conducted in the absence of any commercial or financial relationships that could be construed as a potential conflict of interest.

## Footnotes

1 http://www.addgene.org/

## Supplementary Material

Figure S1 Dissecting microscope image of single colonies formed by Rlv3841[pOPS0754]. **(A)** Bright-field and fluorescence detection with **(B)** GFP filter and **(C)** red filter.
