# Supplementary figures and images for: A Bacterial Expression Vector Archive (BEVA) for Flexible Modular Assembly of Golden Gate-Compatible Vectors
Source: Front Microbiol. 2019 Jan 14;9:3345. doi: 10.3389/fmicb.2018.03345 (PMC6339899; doi:10.3389/fmicb.2018.03345)

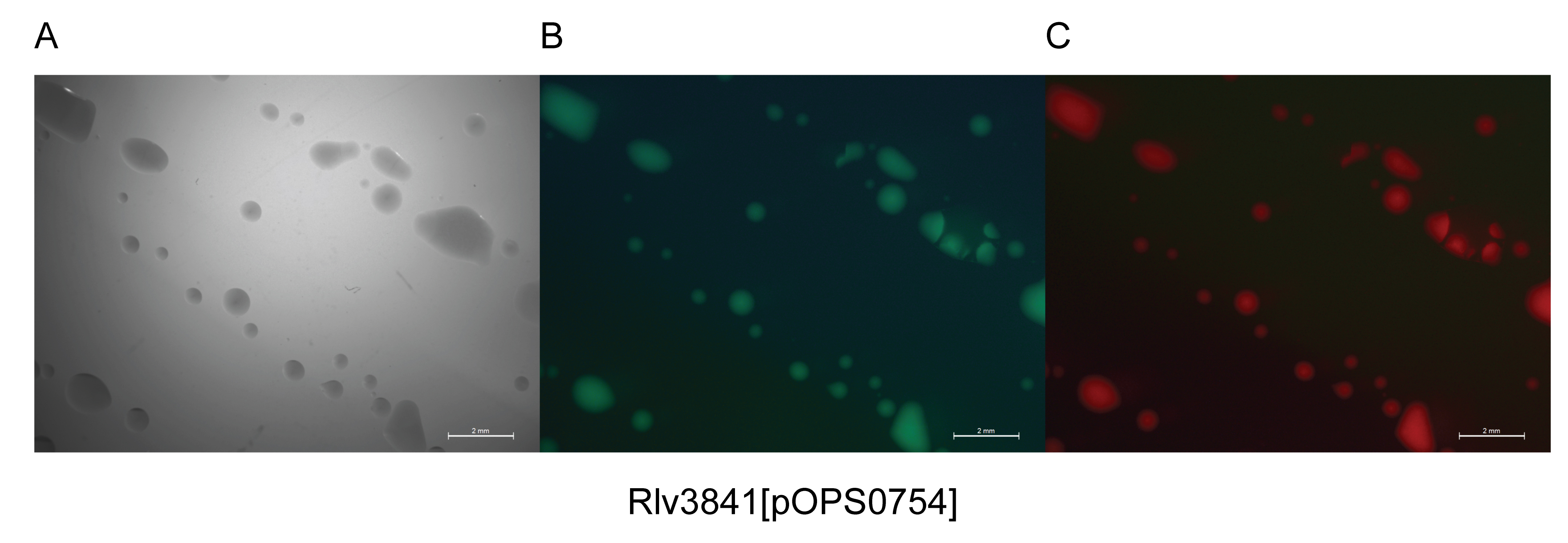

Supplement: Figure S1 — Dissecting microscope image of single colonies formed by Rlv3841[pOPS0754]. (A) Bright-field and fluorescence detection with (B) GFP filter and (C) red filter. [file Image_1.JPEG]
